# Human Immunodeficiency Virus-1 Latency Reversal *via* the Induction of Early Growth Response Protein 1 to Bypass Protein Kinase C Agonist-Associated Immune Activation

**DOI:** 10.3389/fmicb.2022.836831

**Published:** 2022-03-10

**Authors:** Lilly M. Wong, Dajiang Li, Yuyang Tang, Gema Méndez-Lagares, George R. Thompson, Dennis J. Hartigan-O’Connor, Satya Dandekar, Guochun Jiang

**Affiliations:** ^1^UNC HIV Cure Center, Institute of Global Health and Infectious Diseases, The University of North Carolina at Chapel Hill, Chapel Hill, NC, United States; ^2^Department of Medical Microbiology and Immunology, University of California, Davis, Davis, CA, United States; ^3^Department of Biochemistry and Biophysics, The University of North Carolina at Chapel Hill, Chapel Hill, NC, United States

**Keywords:** HIV latency, protein kinase C, EGR1, kick and kill, HIV cure, immune response, HIV reservoir

## Abstract

Human Immunodeficiency Virus-1 (HIV) remains a global health challenge due to the latent HIV reservoirs in people living with HIV (PLWH). Dormant yet replication competent HIV harbored in the resting CD4+ T cells cannot be purged by antiretroviral therapy (ART) alone. One approach of HIV cure is the “Kick and Kill” strategy where latency reversal agents (LRAs) have been implemented to disrupt latent HIV, expecting to eradicate HIV reservoirs by viral cytopathic effect or immune-mediated clearance. Protein Kinase C agonists (PKCa), a family of LRAs, have demonstrated the ability to disrupt latent HIV to an extent. However, the toxicity of PKCa remains a concern *in vivo*. Early growth response protein 1 (EGR1) is a downstream target of PKCa during latency reversal. Here, we show that PKCa induces EGR1 which directly drives Tat-dependent HIV transcription. Resveratrol, a natural phytoalexin found in grapes and various plants, induces *Egr1* expression and disrupts latent HIV in several HIV latency models *in vitro* and in CD4+ T cells isolated from ART-suppressed PLWH *ex vivo*. In the primary CD4+ T cells, resveratrol does not induce immune activation at the dosage that it reverses latency, indicating that targeting EGR1 may be able to reverse latency and bypass PKCa-induced immune activation.

## Introduction

Integration of human immunodeficiency virus-1 (HIV) provirus in the human genome allows HIV to persist in resting CD4+ T cells in people living with HIV (PLWH), known as latent HIV reservoirs. Prolonged use of antiretroviral therapy (ART) mitigates progression of HIV infection; however, it is not a cure as it fails to eradicate latent HIV infected immune cells. Models indicate that long-term ART would need to be sustained for a minimum of 60 years due to the inability to purge the reservoirs (Finzi et al., 1999; Crooks et al., 2015; Siliciano and Siliciano, 2015). Thus, the consequence of discontinuing ART leads to rapid viral rebound, usually within weeks. Therefore, current ART has no impact on the quiescent viral reservoirs due to the extremely low levels of HIV expressed in the immune cells. An approach to directly target latent HIV reservoirs is urgently needed.

A widely explored approach for HIV cure is “kick and kill,” where small molecule agents, also known as latency reversal agents (LRAs), are used to stimulate HIV expression (“kick”) for successive viral cytopathic effects or immune clearance (“kill”) (Archin et al., 2012; Deeks, 2012). Epigenetic reprogramming at the HIV long terminal repeat (LTR) with histone deacetylase inhibitors (HDACi) and NF-κB activation with protein kinase C agonists (PKCa) constitute two of the major classes of LRAs (Abner and Jordan, 2019). Some HDACi such as Vorinostat, Romidepsin, and Panobinostat have advanced into clinical trials or in a clinic setting (Archin et al., 2012; Tsai et al., 2016; Kroon et al., 2020; Rosas-Umbert et al., 2020). Archin et al. (2012) presented a proof-of-concept study for the use of Vorinostat in HIV-infected individuals where increased HIV RNA expression was found in eight participants after a single dose of Vorinostat, providing the first evidence of LRA *in vivo*. However, no reservoir reduction was observed (Archin et al., 2012; Tsai et al., 2016). A study by Kroon et al. (2020) indicated that the time for viral rebound to occur and the overall size of the latent HIV reservoir may not be impacted by Vorinostat administration in ART-suppressed HIV-positive individuals. Previous studies involving PKCa portray robust latency reversal compared with HDACi *in vitro* (Jiang et al., 2014; Stoszko et al., 2019) and, to a lesser extent, *ex vivo* (Jiang et al., 2014, 2015; Pandelo Jose et al., 2014; Darcis et al., 2015; Cary et al., 2016; Spivak and Planelles, 2018; Yang et al., 2019). PKCa and their derivatives have been tested in several pre-clinic models (Gutierrez et al., 2016; Marsden et al., 2017, 2020; Jiang et al., 2019; Okoye et al., 2022), indicating their potential for latency reversal *in vivo.* Some PKCa such as FDA-approved ingenol compounds (PEP005, trademark name: PICATO for topical use) showed potency in the reactivation of latent HIV after topical application to the skin of PLWH on ART (Jiang et al., 2019). However, an initial double-blind Phase 1 trial of bryostatin-1 failed to reactivate latent HIV in ART-suppressed PLWH (Gutierrez et al., 2016). While the low dosages administered were safe in conclusion, further studies are needed to evaluate whether a higher dosage would be effective and safe (Gutierrez et al., 2016). These initial studies indicate that although PKCa are candidates for latency reversal, the use of this group of small molecular agents remains a concern as many of the LRAs act upon host cells and may cause toxicity of immune cells *in vivo* at the dosages that reverse latency (Pardons et al., 2019). PKCa are strong transcriptional activators of PKC/NF-κB, and in turn can cause a cascade of signaling events as an undesired immune response (i.e., T-cell activation and inflammation) (Jiang et al., 2014). Therefore, evaluating the basic mechanism of action by PKCa is necessary to potentiate the use of PKCa *in vivo*. Studies are underway to pursue new derivatives of PKCa, such as ingenol compounds GSK445A and SUW133, to efficiently disrupt latent HIV for eradication with minimal side effects and immune activation *in vivo* (Kim et al., 2022; Okoye et al., 2022), which support the concept that appropriate PKCa can be achieved by synthesizing new PKCa family compounds for the cure of HIV (Wender et al., 2008; Beans et al., 2013; Marsden et al., 2017, 2018, 2020; Jiang et al., 2019; Okoye et al., 2022). Recently, it was discovered that the family compounds of PKCa trigger a conserved downstream signaling of EGR pathway to induce HIV transcription from latency, which was significantly dampened when EGR proteins were knocked down (Vemula et al., 2017). This indicates that EGR protein is the effector of PKCa, in which EGR signaling is an essential molecular mechanism of latency reversal elicited by the family of PKCa. This may represent an alternative approach to disrupt latent HIV. Nevertheless, it is unknown whether EGR proteins directly regulate HIV transcription and whether a direct induction of EGR disrupts latent HIV without the induction of the subsequent immune activation. In this study, we aimed to address these important questions for the role of EGR1 in HIV cure.

## Results

### Early Growth Response Protein 1 Is Highly Correlated With Human Immunodeficiency Virus-1 Transcription or Latency Reversal Induced by Families of Protein Kinase C Agonists

**FIGURE 1:**
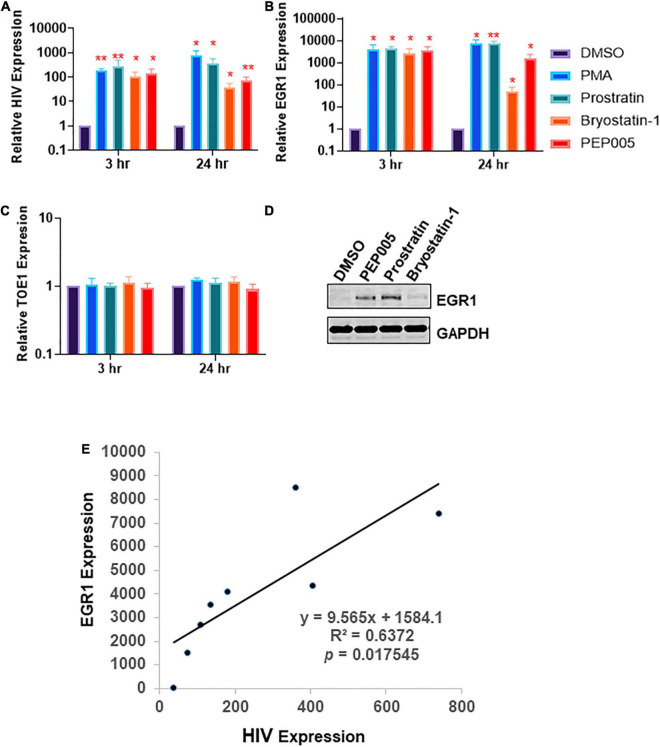
Protein Kinase C agonist (PKCa) response gene, *early growth response protein 1* (*EGR1*), is associated with human immunodeficiency virus-1 (HIV) transcription or latency reversal. **(A–C)** J-Lat A1 cells were treated with 50 ng/mL phorbol 12-myristate 13-acetate (PMA), 2 μM prostratin, 10 nM bryostatin-1 or 12 nM PEP005. Cells were harvested 3 and 24 h post-treatment. The expression of HIV, *EGR1* or *TOE* was analyzed by RT-qPCR (*n* = 3). *p < 0.05; **p < 0.01, compared with control treatment and analyzed by two-tailed *T*-test. Data are represented as mean ± SEM. **(D)** J-Lat A1 cells were treated with 5 μM prostratin, 20 nM bryostatin-1 or 24 nM PEP005. Cells were harvested 4 h post-treatment with PKCa and subjected to Western blot to measure EGR1 protein expression. **(E)** *EGR1* expression and HIV reactivation were plotted to determine linear regression.

We first investigated whether EGR1 was associated with HIV transcription. J-Lat A1, Jurkat cell model of HIV latency, was treated with several PKCa (phorbol 12-myristate 13-acetate (PMA), prostratin, bryostatin-1 and PEP005) at optimized concentrations (Darcis et al., 2015; Jiang et al., 2015) to determine if there was any enhancement of EGR1 expression in HIV reactivated cells (Figure 1). Consistent with previous observations (Pandelo Jose et al., 2014; Darcis et al., 2015; Jiang et al., 2015; Vemula et al., 2017), we found that the commonly used PKCa induced robust HIV expression (up to 1,000-fold) in the J-Lat model of HIV latency whereas at the same time, we observed thousands-fold change in *EGR1* expression (Figures 1A,B). Furthermore, we examined the expression of Target of EGR1 (TOE1), which has been characterized as a direct downstream target of EGR1 and functions as an inhibitor of HIV replication (Sperandio et al., 2015). Surprisingly, *TOE1* expression did not change across the PKCa-treated cells, indicating that TOE1 may not be directly related with EGR1 even though EGR1 was highly induced by PKCa in the Jurkat cells (Figure 1C). To confirm whether EGR1 protein expression was indeed induced by PKCa, whole protein lysates were collected from J-Lat A1 cells that were treated with PEP005, prostratin or bryostatin-1 where PEP005 and prostratin had induced the highest EGR1 expression while bryostatin-1 had minimal impact on EGR1 expression (Figure 1D). Furthermore, we conducted a linear regression analysis of EGR1 expression compared to HIV reactivation where the observed R_2_ value is 0.637 with *p* = 0.0175, suggesting that EGR1 is highly correlated to the triggered HIV reactivation (Figure 1E). EGR1 may serve as the common downstream factor to induce HIV latency reversal prompted by the family of PKCa as discovered previously (Vemula et al., 2017).

**FIGURE 2:**
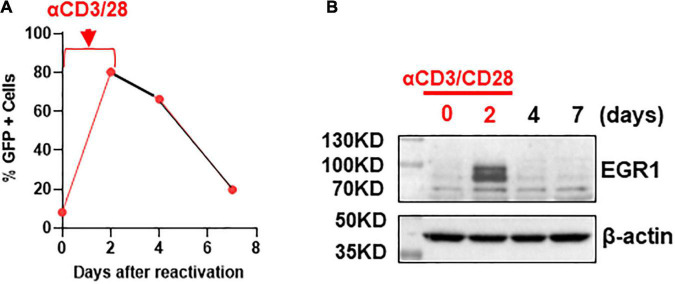
Early growth response protein 1 protein is induced after T cell activation *via* αCD3/CD28 stimulation but waned when HIV re-entered the latent state. **(A)** Transcription of HIV in the primary CD4+ T cell model of HIV latency. The percentage of GFP positive cells was analyzed by flow cytometry at 0, 2, 4, and 7 days (*n* = 3). **(B)** EGR1 protein expression was analyzed in the primary T cell model of HIV latency during the activation of HIV and when HIV returned to latency where β-actin served as a loading control.

If EGR1 is involved in latency reversal, it should be associated with HIV transcription. We tested this hypothesis in a previously reported primary CD4+ T cell model of HIV latency (Bradley et al., 2018). In brief, CD4+ T cells were isolated from HIV-negative donors and were subsequently infected with GFP-tagged NL4.3Δ6 HIV. A few days post-infection, the GFP+ positive cells were sorted by flow cytometry where the GFP+ cells were continued to culture. Then, a second flow cytometry was performed to selectively isolate GFP-negative cells. These GFP-negative HIV genome containing cells represent the latently infected CD4 + T cells where HIV expression can be reactivated by α-CD3/CD28 beads. After the beads were removed, GFP expression waned, indicating the reestablishment of the latent state (Figure 2A). In the process of HIV transcription, EGR1 expression was at its highest at 2 days post-stimulation with α-CD3/CD28 beads where the highest levels of HIV transcription was also observed. Then, it quickly diminished as HIV returned to its latent state (Figure 2B). These data are consistent to previous reports and suggest that EGR1 may be involved in HIV transcription.

### Early Growth Response Protein 1 Directly Induces Tat-Dependent Human Immunodeficiency Virus-1 Transcription

**FIGURE 3:**
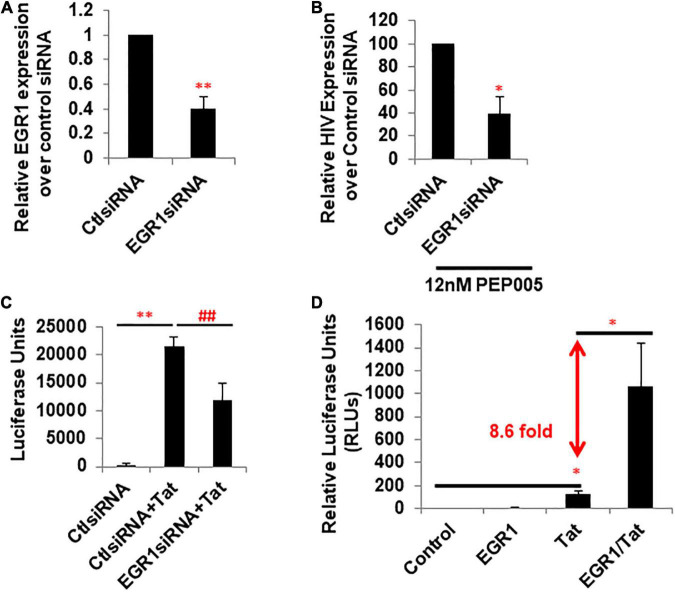
Expression of EGR1 is associated with Tat-dependent HIV transcription. **(A)** TZM-bl cells were transfected with control or EGR1 siRNA. Cells were harvested 48 h post-knockdown and subjected to RT-qPCR (*n* = 3). ^∗∗^*p* < 0.01, compared with control siRNA and analyzed by two-tailed *T*-test. Data are represented as mean ± SEM. **(B)** TZM-bl cells were transfected with control or EGR1 siRNA. Then, these cells were treated with 12 nM PEP005 48 h post-knockdown. Cells were harvested 24 h post-LRA treatment and subjected to RT-qPCR (*n* = 3). ^∗^*p* < 0.05, compared with control siRNA and analyzed by two-tailed *T*-test. Data are represented as mean ± SEM. **(C)** TZM-bl cells were transfected by control or EGR1 siRNA in panel **(B)**, which were then transfected with empty vector or pcDNA-flag-Tat. Cells were harvested after 48 h and subjected to luciferase assay. ^∗∗^*p* < 0.01, compared with control siRNA and analyzed by two-tailed *T*-test. ^##^*p* < 0.01, compared with control siRNA with Tat and analyzed by two-tailed *T*-test. Data are represented as mean ± SEM. **(D)** TZM-bl cells were transfected with empty vector, pcDNA3-EGR1, pcDNA-Flag-Tat, or in combination. Cells were harvested 24 h post-transfection and subjected to luciferase assay. ^∗^*p* < 0.05, compared with control siRNA and analyzed by two-tailed *T*-test.

To see whether EGR plays a direct role in PKCa-induced HIV latency reversal or HIV transcription, we knocked down EGR1 by its specific siRNA in TZM-bl reporter cells. Relative *EGR1* expression compared to the control EGR1 siRNA decreased 2.5-fold (Figure 3A). HIV expression was evaluated in the presence of PEP005, a PKCa to reactivate latent HIV and induce EGR1 (Figures 1, 2), where *EGR1* expression decreased 2.5-fold (Figure 3B). We wanted to determine whether Tat-driven transcription is directly regulated by EGR1 expression. *EGR1* was knocked down in the TZM-bl cells where cells were transfected with plasmid encoding HIV Tat afterward. Transfection of HIV Tat highly induced HIV transcription, which was significantly inhibited when *EGR1* was knocked down (Figure 3C). In the same model, TZM-bl cells were transfected with plasmids encoding EGR1, Tat, or Tat plus EGR1 (Figure 3D). Notably, there was 8.6 fold increase of Tat-induced HIV transcription in the presence of EGR1 plasmids compared with cells transfected with Tat plasmids alone (Figure 3D). Taken together, our data suggest that EGR1 may directly participate in the transcriptional machinery. This may partially explain the role of EGR1 as a conserved downstream effector of PKCa during latency reversal.

### Resveratrol Induces *Early Growth Response Protein 1* Where Early Growth Response Protein 1 Is Recruited to Human Immunodeficiency Virus-1 Long Terminal Repeat to Reactivate Human Immunodeficiency Virus-1 From Latency

**FIGURE 4:**
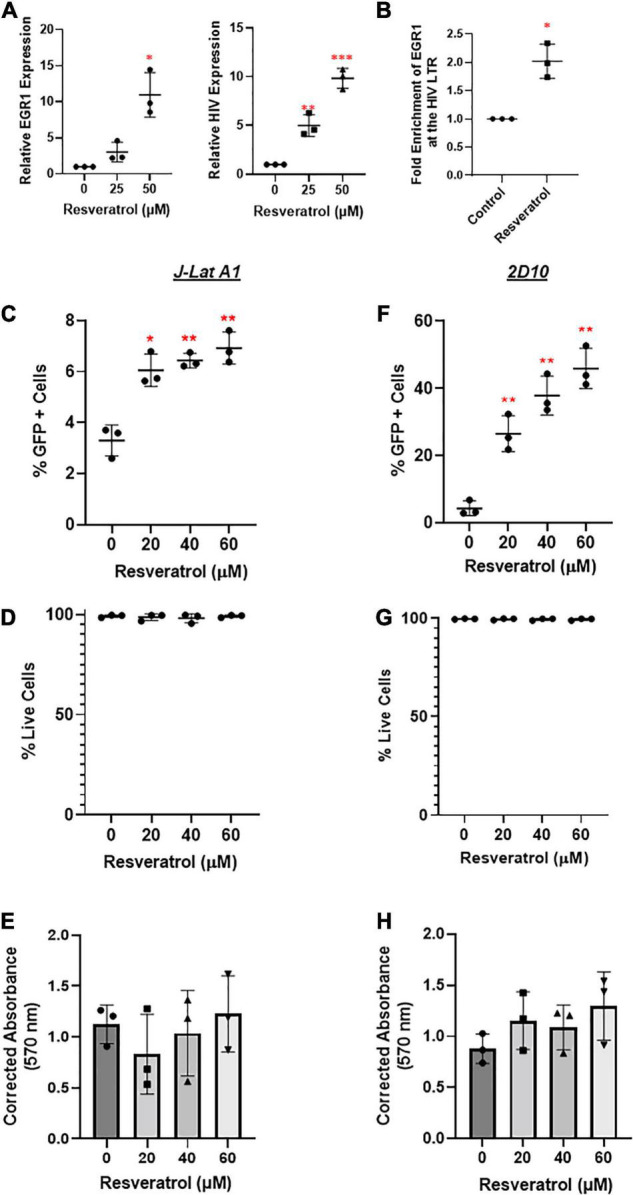
Resveratrol induces *EGR1* expression and reactivates latent HIV *in vitro*. **(A)** J-Lat A1 cells were treated with resveratrol at 25 and 50 μM. Cells were harvested for RT-qPCR analysis (*n* = 3) 24 h post-treatment. *p < 0.05; **p < 0.01, ***p < 0.001 compared with control treatment and analyzed by two-tailed *T*-test. **(B)** J-Lat A1 cells were treated with resveratrol at 40 μM for 24 h. Cells were harvested and cross-linked with 1% formaldehyde to prepare chromatin. ChIP assay was performed with control IgG and anti-EGR antibodies. The enrichment of EGR1 at the HIV promoter was measured by SYBR green PCR with the specific primers targeting HIV long terminal repeat (LTR) region. *p < 0.05; compared with control treatment and analyzed by two-tailed *T*-test. J-Lat A1 **(C–E)** and 2D10 **(F–H)** cells were treated with resveratrol (20, 40, and 60 μM). The percentage of GFP-positive cells and cell viability were analyzed by flow cytometry 24 h post-treatment while the cell proliferation was measured by 3-(4,5-dimethylthiazol-2-yl) -2,5-diphenyltetra- zolium bromide (MTT) assay. *p < 0.05; **p < 0.01, compared with control treatment and analyzed by two-tailed *T*-test (*n* = 3).

Our studies above point to an interesting concept: the induction of EGR1 may be able to drive HIV transcription from latency, which could bypass the undesired side effects PKCa may elicit (French et al., 2020). Resveratrol has been described as an inducer of EGR1, which is involved in HIV transcription (Krishnan and Zeichner, 2004). However, its underlying mechanism is unclear. To evaluate the potential of resveratrol as an EGR1 agonist (EGRa), we first tested its activity in several models of HIV latency. We tested several concentrations of resveratrol and observed *EGR1* induction in a dose-dependent manner in J-Lat A1 cells (Figure 4A). Importantly, this also induced EGR1 enrichment at the HIV LTR (Figure 4B). GFP expression, a measure of HIV transcription at the protein level, increased in a dose-dependent manner (Figures 4C,F). In both J-Lat A1 and 2D10 cell models of HIV latency, no toxicity or minimal impact of cell proliferation was observed even at the highest tested concentration (60 μM) (Figures 4D,E,G,H). These data suggest that EGR1, after induction by resveratrol, may serve as a HIV transcription factor after its recruitment to HIV promoter to induce HIV transcription and/or latency reversal.

### Resveratrol Serves as a Stimulator to Enhance Human Immunodeficiency Virus-1 Transcription Induced by Other Latency Reversal Agents

**FIGURE 5:**
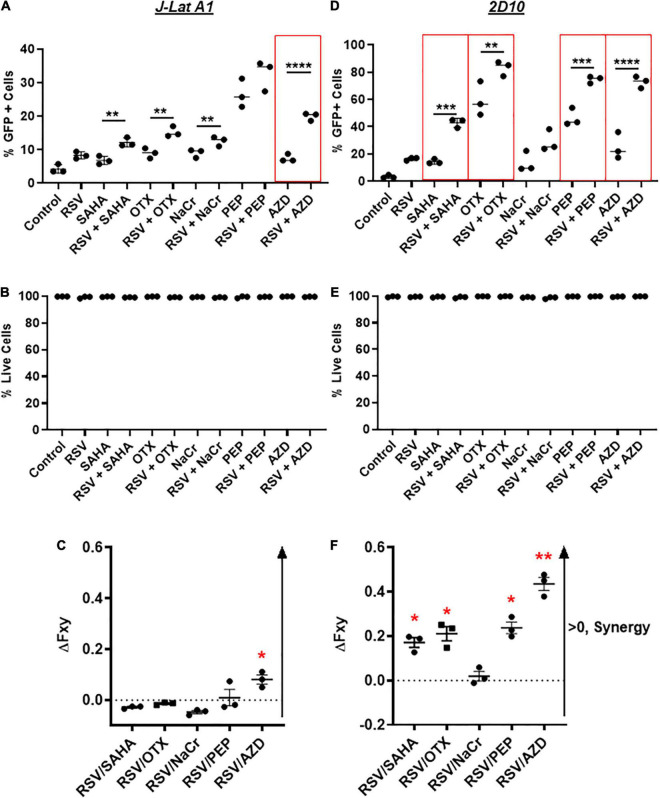
Resveratrol enhances latency reversal in combination with AZD5582 and other established LRAs *in vitro*. J-Lat A1 cells **(A,B)** and 2D10 cells **(D,E)** were treated with 40 μM resveratrol (RSV), 500 nM suberoylanilide hydroxamic acid (SAHA), 10 nM OTX-015 (OTX), 40 mM crotonate (NaCr), 12 nM PEP005, 10 nM AZD5582 or in combination with 40 μM resveratrol. The percentage of GFP-positive cells and cell viability were analyzed by flow cytometry 24 h post-treatment (*n* = 3). **p < 0.01; ***p < 0.001; ****p < 0.0001, compared with single LRA treatment and analyzed by One-way ANOVA. Bliss independence analysis was performed to evaluate the synergistic effect of resveratrol with other LRAs in comparison with the single LRA in J-Lat A1 **(C)** and 2D10 **(F)** cells. *p < 0.05; **p < 0.01, compared between faxyP and faxyO by two-tailed *t*-test analysis.

Current LRAs alone may not be potent enough to disrupt latent HIV and/or present the viral antigen for the subsequent killing of HIV+ immune cells (Margolis et al., 2016; Hosmane et al., 2017; Dashti et al., 2020). To this end, we then decided to test whether resveratrol enhances HIV reactivation when combined with other known LRAs such as AZD5582, SAHA, OTX-015 and PEP005. We focused on these LRAs because: (1) it has been shown that PKCa has the best synergy with bromodomain inhibitors to disrupt latent HIV (Darcis et al., 2015; Jiang et al., 2015); (2) OTX-015, a bromodomain inhibitor, is able to disrupt latent HIV, and is under clinical trials for the cure of cancer (Lu et al., 2016; Yin et al., 2020); (2) SAHA and crotonate (NaCr) are epigenetic inducers (Archin et al., 2012; Jiang et al., 2018). SAHA and other HDACi have been tested in a clinic and/or pre-clinic setting; (3) Unlike PKCa-driven canonical NF-κB (cNF-κB) signaling, AZD5582 is a new generation of LRA that drives non-canonical NF-κB (ncNF-κB) signaling activation which displayed potential as a LRA in animal models (Nixon et al., 2020). We aimed to see whether a robust enhancement of latency reversal can be achieved. In order to achieve optimal latency reversal without triggering cellular toxicity, 40 μM of resveratrol was used during the combination treatment (Figure 4). Overall, we observed the enhancement of HIV transcription in most of those combinations compared with single LRA, except for PEP005 in combination with resveratrol in J-Lat A1 and NaCr in 2D10 cells (Figures 5A,D). No toxicity was observed in either J-Lat A1 or 2D10 cells (Figures 5C,E). When analyzed with Bliss independence assay, among all the other combinations, only the combination of AZD5582 with resveratrol showed a synergy in both J-Lat A1 and 2D10 cell models of HIV latency (Figures 5D,F). These data further support an idea that combination treatment may be better to induce HIV from latency where the combination of resveratrol with the activation of ncNF-κB by AZD5582 displays the optimal latency reversal.

### Resveratrol Induces Human Immunodeficiency Virus-1 Transcription in the Primary CD4+ T Cells Isolated From Antiretroviral Therapy-Suppressed People Living With Human Immunodeficiency Virus-1

**FIGURE 6:**
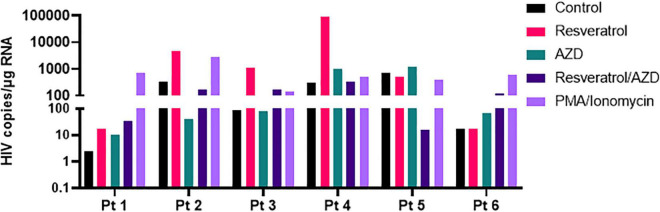
Resveratrol reactivates latent HIV in the primary CD4+ T cells isolated from ART-suppressed PLWH. Total CD4+ T cells from ART-suppressed PLWH were treated with 40 μM resveratrol, 100 nM AZD5582 (AZD), 200 ng/mL PMA/2 μM ionomycin or in combination with RSV and AZD. Cells were harvested 24 h post-treatment, and copies of cell-associated HIV RNA were analyzed by ddPCR after normalization to the reference gene TBP.

Often times, LRAs can disrupt latent HIV *in vitro* but fails to recapitulate in patient cells *ex vivo* and/or *in vivo*, reflecting the extreme difficulty to eradicate HIV reservoirs in patients (Grau-Exposito et al., 2019). To evaluate the potential of resveratrol as an EGRa, we continued to test its activity in the primary CD4+ T cells isolated from PLWH receiving ART (Table 1). We aimed to test both its activity alone and in combination with AZD5582 since only the combination of resveratrol with AZD5582 showed synergy in both of the HIV latency models *in vitro*, compared with other combinations (Figure 5). We found that resveratrol alone was able to induce HIV transcription from latency in 4 of 6 samples while AZD5582 alone also disrupted latent HIV in 4 of 6 samples. Although PMA/ionomycin is among one of the more potent LRAs, it failed to induce HIV transcription in 2 of 6 samples. Different from latency models *in vitro*, combination treatment only enhanced latency reversal in 2 of 6 samples of primary CD4 + T cells isolated from PLWH. These data suggest that EGRa may be able to induce HIV transcription from latency in some, but not all, of the patient immune cells. Different from *in vitro* cell line model of latency, less samples achieved enhanced latency reversal. These observations further strengthen the idea that the latent HIV is extremely hard to disrupt and CD4 + T cells from PLWH may establish latency differently (Figure 6).

**TABLE 1:** Patient information in this study.

Patient ID	Age	Sex	Viral load (copies/ml)	CD4 count (cells/μl)	Duration of ART (years)	ART regimen
1	33	M	Undetectable	854	1	Triumeq
2	60	M	Undetectable	742	19	Triumeq
3	33	M	Undetectable	681	21	Odefsey
4	39	M	30	826	20	Triumeq
5	68	M	Undetectable	376	31	Triumeq
6	56	M	47	412	25	Ritonavir/ Darunavir/ Raltegravir

### Resveratrol Does Not Induce Immune Activation in the Primary CD4+ T Cells Isolated From Human Immunodeficiency Virus-1 Negative Donors

**FIGURE 7:**
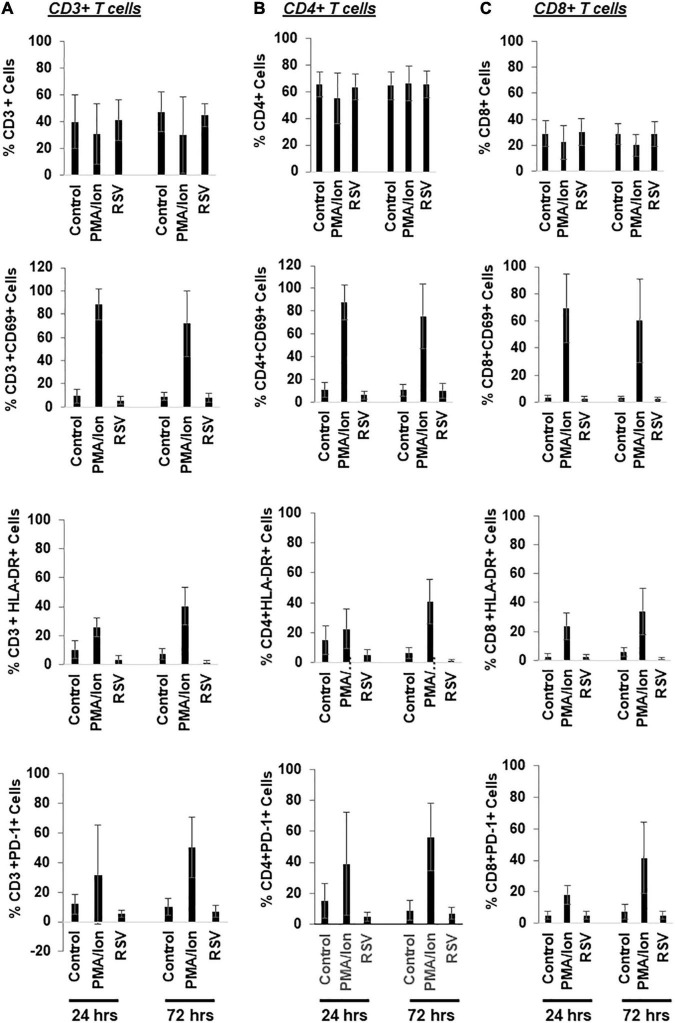
Resveratrol bypasses immune activation in the primary CD4+ T cells isolated from HIV negative donors. **(A–C)** PBMCs from healthy donors were treated with DMSO, 40 μM resveratrol (RSV) or 200 ng/mL PMA plus 2 μM ionomycin for 24 or 72 h. Cells were then collected for flow cytometry analysis. Percentage of immune activation/suppression markers in T cell subsets (CD3+, CD4+, CD8+, and PD-1+) was analyzed by flowjo (*n* = 5).

Resveratrol may be able to disrupt latent HIV *in vitro* and *ex vivo* (Figures 3, 6). We next decided to examine its impact in immune activation. We evaluated this in the CD3+, CD4+ and CD8+ subsets of T cells in the PBMCs from HIV negative donors (Figure 7). We found that resveratrol did not change the percentage of the CD3+, CD4+, or CD8+ T cells, nor did it trigger CD69 and HLA-DR expression across these cells. PD-1 expression was unchanged in these resveratrol-treated cells compared to control-treated cells. These data suggest that resveratrol may not significantly induce immune activation.

## Discussion

Current strategies of HIV cure are faced with the challenge of latent HIV reservoirs, which are extremely difficult to disrupt. “Kick and kill” flushes out the hidden HIV from the latently infected CD4+ T cells for immune clearance (Deeks, 2012). Among the currently available and highly active LRAs are PKCa (Wender et al., 2008; DeChristopher et al., 2012; Jiang et al., 2015; Cary et al., 2016; Lu et al., 2016; Wang et al., 2017). However, there remains concerns of toxicity when using PKCa due to the potential for cytokine storms induced by cNF-κB activation, indicating a need to further study the mechanisms of action involved in PKCa. Due to these concerns, alternative approaches are being pursued, including IAP inhibitors to activate ncNF-κB signaling (Nixon et al., 2020; Wong and Jiang, 2021; Li et al., 2022), synthesis of a new generation of PKCa through analog development (Wender et al., 2006; Jiang et al., 2014; Marsden et al., 2018; Okoye et al., 2022), or exploiting Chinese traditional medicine such as Kansui (Jiang et al., 2014; Cary et al., 2016; Wang et al., 2017). Here, we evaluated an alternative approach to circumvent cNF-κB activation by PKCa by directly activating its downstream effector protein, EGR1, and assessed its related side effects of immune activation.

Previous studies have shown that EGR family proteins serve as the common downstream effectors of PKCa during its action to disrupt latent HIV (Vemula et al., 2017). EGR knockdown prevented robust latency reversal when treated with PKCa (Vemula et al., 2017). Therefore, it may be useful to find a compound that can induce EGR family protein to force HIV transcription from latency. Our data showed the strong correlation between *EGR1* expression and HIV reactivation in several cell models of HIV latency where active transcription was responsive to the peak EGR1 expression. When *EGR1* is knocked down, PEP005-induced HIV transcription or latency reversal was reduced. This is consistent to previous observations (Vemula et al., 2017). Importantly, our data indicate a direct role of EGR1 in HIV transcription since EGR1 was recruited to HIV LTR during latency reversal by EGRa. It can directly drive Tat-dependent HIV transcription. It is possible that EGR1 serves as a transcription factor to directly or indirectly interact with HIV LTR for its action. However, the underlying molecular mechanism is unclear, which warrants further investigation in future.

Resveratrol, a natural phytoalexin commonly found in red wine, has been used as a preventative measure for cardiovascular disease (Zeng et al., 2017; Cheng et al., 2020). Due to its naturally occurring state, others are investigating whether it could be repurposed for other disease treatments (Zeng et al., 2017). Interestingly, resveratrol has been implicated in the disruption of HIV latency; however, the mechanism is unclear, nor can it disrupt latent HIV in the patient-derived CD4+ T cells (Sampey et al., 2020). Our data showed that resveratrol induces *EGR1* expression and can reactivate latent HIV in several cellular models of HIV latency. This was observed in the majority of, but not all, the samples of the CD4 + T cells isolated from PLWH on ART. It has been shown that resveratrol induces histone acetylation to drive HIV transcription (Zeng et al., 2017). However, we failed to detect the activity of acetylation at H3K9 at the dosage of latency reversal in the Jurkat model of HIV latency (Supplementary Figure 1). It is worth pointing out that resveratrol may have some the other signaling targets, in addition to EGR1 (Tsai et al., 2017).

Together, our data support an idea that it is possible to develop an alternative approach to directly activate EGR1, the common downstream effector of PKCa, by EGRa to disrupt latent HIV, avoiding PKC/cNF-κB-induced immune activation. While EGR1 serves as an alternative therapeutic target to reverse HIV latency, studies are needed to develop a potent and specific inducer of EGR1 to reverse HIV latency in patient immune cells.

## Materials and Methods

### Cell Culture

J-Lat A1 and 2D10 cell models of HIV latency were maintained in RPMI 1640 medium with 10% fetal bovine serum (Avantor, Allentown, PA, United States), 1% penicillin-streptomycin (Gibco, Waltham, MA, United States), and 1% L-glutamine (Gibco) in 37°C incubator with 5% CO_2_. TZM-bl cells were maintained in DMEM medium with 10% fetal clone II serum (FCS II, Cytiva, Marlborough, MA, United States), 1% penicillin-streptomycin (Gibco), 1% HEPES (Gibco), and 1% L-glutamine (Gibco). TZM-bl is a HIV transcription/replication reporter cell line derived from HeLa where it constitutively expresses CD4, CXCR4, and CCR5. The HIV-Tat responsive TZM-bl cell line includes firefly luciferase reporter gene where the enzymatic output can be used to enlighten our understanding of HIV. These cell lines were provided by the National Institutes of Health (NIH) AIDS Reagent Program. The following reagents were used to test the reactivation of latent HIV: dimethyl sulfoxide (Sigma-Aldrich, St. Louis, MO, United States), phorbol 12-myristate 13-acetate (PMA) (Sigma-Aldrich), prostratin (Sigma-Aldrich), bryostatin-1 (Tocris Bioscience, Bristol, United Kingdom), PEP005 (Tocris Bioscience), resveratrol (RSV) (Celbiochem, San Diego, CA, United States) suberoylanilide hydroxamic acid (SAHA) (Santa Cruz, CA, United States), OTX-015 (Xcess Bioscience Inc., San Diego, CA, United States), crotonate (Sigma) and AZD5582 (ChemieTek, Indianapolis, IN, United States).

### Human Immunodeficiency Virus-1 Gene Expression Measurement by Real-Time Quantitative PCR

Cellular RNA was extracted by RNeasy mini kit (Qiagen). Extracted RNA was digested by DNase 1 (Invitrogen, Waltham, MA, United States) where first-strand cDNA was synthesized with SuperScript™ III Reverse Transcriptase (Invitrogen), dNTPs (Qiagen, Germantown, MA, United States), 0.1 M DTT (Invitrogen), Ribolock RNase Inhibitor (Thermo Scientific, Waltham, MA, United States) and Random Primers (Invitrogen) where it was carried out in Veriti™ 96-Well Thermal Cycler (Applied Biosystems, Waltham, MA, United States). HIV reactivation was quantified by Taqman quantitative RT-PCR (RT-qPCR) on the QuantStudio 5 system (Applied Biosystems) with HIV pre-gag primers/probe set as previously described (Jiang et al., 2015). Housekeeping genes, glyceraldehyde-3-phosphate dehydrogenase (GAPDH) and TATA-box binding protein (TBP) primers/probe sets were used for endogenous controls (Applied Biosystems).

### Evaluation of Human Immunodeficiency Virus-1 Reactivation by Flow Cytometry

Cells were cultured in 12-well plates at 1 × 10^6^ cells/well. Cells were treated with LRAs. Twenty four hours post-treatment, cells were measured for endogenous GFP expression and cell viability by flow cytometry. Cells were stained with LIVE/DEAD Fixable Far Red stain (Invitrogen) to quantify cell viability by flow cytometry. Flow cytometry results were analyzed by FlowJo™ v10.8 Software (BD Life Sciences, Franklin Lakes, NJ, United States).

### 3-(4,5-Dimethylthiazol-2-yl)-2,5-Diphenyltetrazolium Bromide Assay to Determine Cellular Viability/Proliferation After Latency Reversal

Cells were cultured in 96-well plates at 5 × 10^4^ cells/well in 100 μl culture medium and treated with LRAs. To assess proliferation of the cells, CellTiter 96 Non-Radioactive Cell Proliferation Assay (Promega, Madison, MI, United States) was used. Twenty-four hours post-treatment, 15 μl of the Dye Solution was added to each well. The plate was incubated at 37°C for another 4 h. After incubation with the Dye Solution, 100 μl of the Solubilization Solution/Stop Mix was added to each well. One hour after addition of the Solubilization Solution/Stop Mix, the absorbance was measured at 570 nm wavelength with Synergy H1 (BioTek, Winooski, VY, United States). Absorbance values of samples were subtracted from the average reference absorbance values to determine the corrected absorbance value at 570 nm for each sample.

### Western Blot

Cells were lysed in RIPA buffer (Sigma-Aldrich) supplemented with 1X Protease/Phosphatase Inhibitor Cocktail (Cell Signaling, Danvers, MA, United States) and separated on 4–20% gradient SDS/PAGE (Novex). Proteins were transferred on nitrocellulose membranes and incubated with primary antibodies overnight at 4°C. The primary antibodies used for Western blotting were rabbit anti-EGR1 (#4153S; Cell Signaling), rabbit anti-GAPDH (#5174S; Cell Signaling) and rabbit anti-β-actin (#4970S; Cell Signaling). The secondary antibody used was HRP linked anti-rabbit IgG (#7074P2; Cell Signaling). Chemiluminescence (ECL) was used to detect protein abundance.

### Primary CD4+ T Cell Model of Human Immunodeficiency Virus-1 Latency

The primary CD4 + T cell model of latency was used as described (Bradley et al., 2018). Cells were reactivated with αCD3/CD28 beads for 2 days which were then harvested for protein in RIPA buffer (Sigma-Aldrich) supplemented with protease/phosphatase inhibitors cocktail (Cell Signaling). Protein expression of EGR1, GAPDH, or β-actin was evaluated. Cells were also harvested for the evaluation of GFP expression by flow cytometry at 0, 2, 4, and 7 days.

### Gene Knockdown by Small Interfering RNA and Transfections

Gene knockdown was achieved by Human EGR1 siRNA (E-006526-00-0005, Dharmacon) or scrambled control siRNAs (D-001910-10-20, Dharmacon) in TZM-bl cells. Cells were incubated with the siRNAs for 48 h where cells were then re-suspended in DMEM medium with 10% fetal bovine serum (VWR), 1% penicillin/streptomycin (Gibco), 1% L-glutamine (Gibco), and 1% HEPES (Gibco) for 24 h. Cells were harvested where RNA was extracted to check relative Egr1 expression in TZM-bl cells. To observe whether LRAs impact HIV expression in the case of Egr1 knockdown, cells were treated with 12 nM PEP005. RNA was extracted from the treated cells to check relative HIV expression. The expression plasmid, pcDNA-Flag-Tat, was transfected into TZM-bl cells after Egr1 knockdown. Cells were harvested for luciferase to determine impact. To determine whether Egr1 synergized with Tat, cells were transfected with pCDNA3.1, pcDNA3-Egr1, pcDNA-Flag-Tat, or in combination. Cells were harvested for luciferase activity.

### Luciferase Assays

Luciferase quantification was completed according to manufacturer’s instructions for the Luciferase Assay System (Promega).

### Chromatin Immunoprecipitation

In total, 5 × 10^6^ J-Lat A1 cells were treated with RSV. Twenty four hours post-treatment, protein-DNA bound in the cells were fixed in 1% formaldehyde for 10 min where the crosslinking was then quenched with 0.125 M glycine for 5 min. Cells were washed 3 times in cold PBS where cell pellets were stored in −80°C. Frozen, cross-linked pellets were re-suspended in ice-cold cell lysis buffer (Lysis Buffer 1) [50 mM HEPES–NaOH (pH 7.5); 140 mM NaCl; 1 mM EDTA; 10% glycerol; 0.5% NP-40; 0.25% Triton X100; protease/phosphatase inhibitor cocktail] for 10 min. After centrifugation (2,700 × *g*; 5 min; 4°C), the nuclear pellet was re-suspended in ice-cold nuclei swelling buffer (Lysis Buffer 2) [10 mM Tris–HCl (pH 8.0); 200 mM NaCl; 1 mM EDTA; 0.5 mM EGTA] for 5 min. After centrifugation (2,700 × *g*; 5 min; 4°C), the nuclear pellets were gently re-suspended in Lysis Buffer 3 [10 mM Tris–HCl (pH 8.0)]. After centrifugation (2,700 × *g*; 5 min; 4°C), the nuclei were re-suspended in 150 μl sonication buffer [0.1% SDS; 1 mM EDTA; 10 mM Tris–HCl (pH 8.0); protease/phosphatase inhibitor cocktail]. The nuclear lysates were sonicated with Bioruptor Pico (Diagenode) to obtain 300–1,000 bp chromatin. Nuclei fragments were collected and diluted in ChIP dilution buffer [2 mM EDTA; 5 mM Tris–HCl (pH 8.0); 1% NP-40; 150 mM NaCl; protease/phosphatase inhibitor cocktail). Approximately 100 μg of DNA was incubated overnight at 4°C with rabbit isotype control IgG (Cell Signaling) and rabbit anti-EGR1 (#4153S; Cell Signaling). Immune complexes were incubated with Dynabeads Protein G under rotation for 2 h at 4°C. Following washes with low salt wash buffer, high salt wash buffer, IP wash buffer and TE wash buffer, immunoprecipitation was eluted in 100 μl elution buffer (1% SDS, 0.1 M NaHCO_3_). Immunoprecipitation was incubated with RNase A, proteinase K, then 5 M NaCl for reverse-crosslinking step overnight. DNA was recovered by ChIP Clean and Concentrator (Zymo, Irvine, CA, United States). Immunoprecipitated DNA was quantitated by SYBR green qPCR targeting HIV LTR region of HIV genome.

### Primary CD4^+^ T Cell Isolation, Treatment, and Digital Droplet PCR Assays

Primary CD4+ T cells were isolated with EasySep Human CD4+ T cell enrichment kit (Stemcell Technologies; Vancouver, BC, Canada). Primary CD4+ T cells were plated at 1 × 10^6^ cells/well and treated with DMSO, resveratrol (40 μM), AZD (100 nM), resveratrol and AZD and PMA (200 ng/ml)/ionomycin (2 μM). Twenty four hours post-treatment, cells were harvested for RNA extraction by RNeasy mini kit (Qiagen). RNA copy was measured by digital droplet PCR with pre-gag primer/probe sequences (Yukl et al., 2018). All participants were provided informed consent, and the study was approved by the UC Davis Institutional Review Board. The patient information has been included in Table 1.

### Bliss Independent Analysis

Synergy of resveratrol with other LRA in latency reversal was determined by the Bliss independence analysis (Laird et al., 2015; Jiang et al., 2018). % of GFP+ cells was normalized with that in PMA-treated cells. For drugs x and y, the predicted fraction in the cells with combination treatment (faxyP) is calculated as the equation faxyP = (fax + fay) − (fax) (fay). The observed combination value (FaxyO) is when the cells were treated by both drug x and drug y together. Then, Δfaxy, i.e., faxyO–faxyP, was defined as synergy if Δ faxy > 0, additive effect if Δ faxy = 0, or antagonism if Δ faxy < 0. Statistical significance was determined using a two-tailed *t*-test between faxyO and faxyP, where ^∗^*p* < 0.05 was considered significant.

### Statistical Analysis

Statistical analysis was performed with Prism GraphPad 9.1. Means and standard errors were calculated for all data points from at least three independent experiments. Statistical significance was determined with One-way ANOVA or two-tailed student’s *t*-test, where *p* value <0.05 was considered significant.

## Data Availability Statement

The raw data supporting the conclusions of this article will be made available by the authors, without undue reservation.

## Author Contributions

GJ conceived the study. LW, DL, and YT performed the experiments in latency models *in vitro and ex vivo*. GM-L and DH-O’C analyzed the phenotypes of primary T cell subsets. GT coordinated the patient blood samples. SD supported the initial study. LW and GJ assembled the data and wrote the manuscript. All authors contributed to the article, read, and revised initial draft and approved the manuscript.

## Conflict of Interest

The authors declare that the research was conducted in the absence of any commercial or financial relationships that could be construed as a potential conflict of interest.

## Publisher’s Note

All claims expressed in this article are solely those of the authors and do not necessarily represent those of their affiliated organizations, or those of the publisher, the editors and the reviewers. Any product that may be evaluated in this article, or claim that may be made by its manufacturer, is not guaranteed or endorsed by the publisher.

## Supplementary Material

Supplementary Figure 1 Resveratrol does not mediate histone acetylation in cell model of HIV latency. 2D10 cells were treated with varying concentrations of resveratrol (20, 40, 60, and 80 μM) where the treatment of 500 nM suberoylanilide hydroxamic acid (SAHA) served as histone acetylation control. Total histone proteins were extracted and histone acetylation at the 9th lysine residue of histone H3 protein (H3K9Ac) was visualized by Western blot with the total histone H3 proteins (Total H3) as the loading control.
