# Supplementary figures and images for: Human Immunodeficiency Virus-1 Latency Reversal via the Induction of Early Growth Response Protein 1 to Bypass Protein Kinase C Agonist-Associated Immune Activation
Source: Front Microbiol. 2022 Mar 10;13:836831. doi: 10.3389/fmicb.2022.836831 (PMC8960990; doi:10.3389/fmicb.2022.836831)

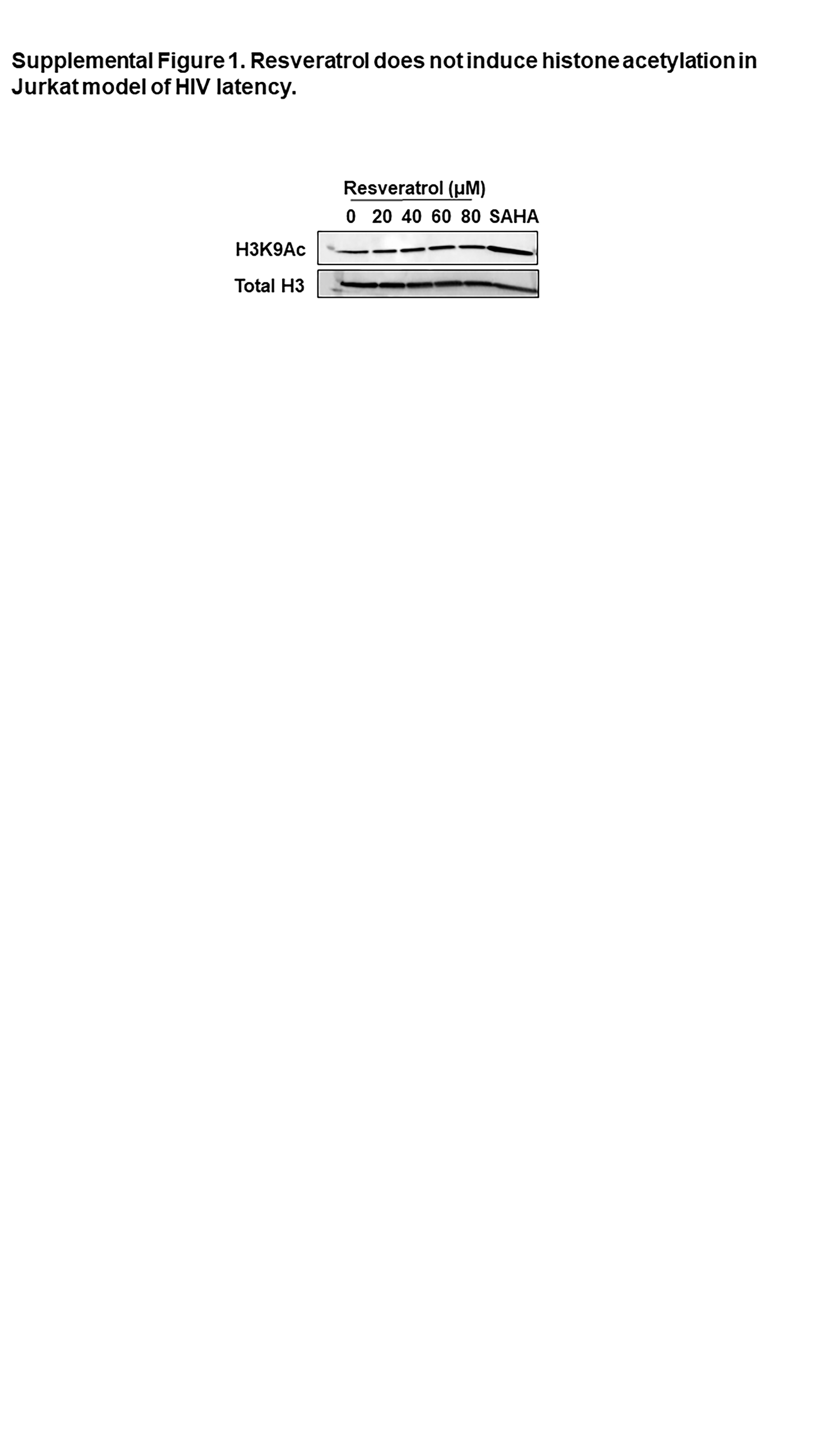

Supplement: Supplementary Figure 1 — Resveratrol does not mediate histone acetylation in cell model of HIV latency. 2D10 cells were treated with varying concentrations of resveratrol (20, 40, 60, and 80 μM) where the treatment of 500 nM suberoylanilide hydroxamic acid (SAHA) served as histone acetylation control. Total histone proteins were extracted and histone acetylation at the 9th lysine residue of histone H3 protein (H3K9Ac) was visualized by Western blot with the total histone H3 proteins (Total H3) as the loading control. [file Image_1.TIF]
